# An adult cystic fibrosis patient presenting with persistent dyspnea: case report

**DOI:** 10.1186/1471-2466-6-9

**Published:** 2006-05-08

**Authors:** Gary M Onady, Catherine L Farinet

**Affiliations:** 1 Medicine-Pediatrics Program, Boonshoft School of Medicine, Wright State University, Suite 500 Elizabeth Place, Dayton, OH, 45408, USA; 2 Piketon Medical Center, 10 Indian Ridge Drive, Suite 1, Piketon, OH 45661, USA

## Abstract

**Background:**

Persistent dyspnea is a common finding in the cystic fibrosis patient that typically leads to further work up of an alternative pulmonary etiology. Adult cystic fibrosis patients; however, are growing in numbers and they are living into the ages in which coronary artery disease becomes prevalent. Coronary disease should be included in the consideration of diagnostic possibilities.

**Case presentation:**

A 52-year-old white male with cystic fibrosis was evaluated for exertional dyspnea associated with vague chest discomfort. Diagnostic testing revealed normal white blood cell, hemoglobin and platelet count, basic metabolic panel, fasting lipid profile, HbA1c, with chest radiograph confirming chronic cystic findings unchanged from prior radiographs and an electrocardiogram that revealed sinus rhythm with left anterior fascicular block. Stress thallium testing demonstrated a reversible anteroseptal perfusion defect with a 55% left ventricular ejection fraction. Heart catheterization found a 99% occlusion of the left anterior descending artery extending into the two diagonal branches, with 100% obstruction of the left anterior descending artery at the trifurcation and 70% lesion affecting the first posterior lateral branch of the circumflex artery.

**Conclusion:**

This case report represents the first description in the medical literature of a cystic fibrosis patient diagnosed with symptomatic coronary artery disease. Applying a standard clinical practice guide proved useful toward evaluating a differential diagnosis for a cystic fibrosis patient presenting with dyspnea and chest discomfort.

## Background

Cystic Fibrosis (CF) is the most common lethal inherited disease in the Caucasian population. It was once considered a childhood disease; however, with advances in health care there is a growing population of adults with CF [1]. A life expectancy that was only 8 years of age in 1974 had advanced to the age of 21 years in 1994, and today is estimated as high as 40 years of age [2]. There are patients on the Cystic Fibrosis Foundation Patient Registry in 2003 that are in their eighth decade of life [1].

With new found longevity comes a new spectrum of disease prevalence associated with aging. Cystic Fibrosis Related Diabetes (43% prevalence) is now the number two chronic illness in the adult CF patient following chronic lung disease and surpassing liver disease (24% prevalence) in patients greater than 30 years of age [3]. Cardiovascular disease has been essentially isolated to cor pulmonale as a consequence of end stage obstructive pulmonary disease. Hypertension has not been considered a serious problem in this patient population [4]; however, a 20% prevalence has been observed at our CF adult center. Symptomatic coronary artery disease, one of the most prevalent of diseases in the adult patient population, has never been reported in the CF population from a PubMed literature search to date. Guides for care of health concerns in adult patients with CF were published in a January 2004 Consensus Report to help transition CF health care from pediatrician to internists or other adult care providers [3]. Many aspects of cardiovascular discussions such as hypertension and forms of heart disease separate from cor pulmonale are missing from this consensus report. The following discussion illustrates the need to continually update and add new information that will lead to optimizing the care of adult CF patients.

## Case presentation

A 52-year-old white male with CF presented with persistent exertional dyspnea and cough with scant sputum production. Physical examination demonstrated oxygen saturation of 91%, with normal temperature and vital signs. His weight had fallen from 79.2 to 77.1 kg over 3 months. Cardiovascular exam was entirely normal. Lungs demonstrated coarse breath sounds bilaterally with scattered rales throughout the lung fields. The abdomen was normal, extremities demonstrated significant clubbing and there was no peripheral edema. Forced expiratory volume at 1 second (FEV_1_) had decreased from 50% to 36%. The chest radiograph resembled baseline findings with no obvious infiltrate or pneumothroax. He received a month-long course of azithromycin, aztreonam and inhaled tobramycin for suspected pulmonary exacerbation of acute super-infection in the setting of underlying chronic CF lung disease.

One month later, follow-up revealed no improvement in dyspnea and no change in the scant sputum production, despite full adherence to the antibiotic regimen and airway clearance techniques. Exertional discomfort located along the sternum and left anterior chest associated with dyspnea without radiation or pressure sensation was further described but was not associated with palpitations, diaphoresis or nausea. This discomfort improved with rest and was not associated with meals. He did report the need to sleep upright in a recliner, but denied paroxysmal nocturnal dyspnea.

CF was diagnosed in this patient by sweat chloride at age 35 after an episode of hemoptysis, with subsequent genetic analysis identifying a ΔF508, 2789+5G>A mutation. He also has gastroesophageal reflux, Barrett's esophagus, azoospermia, and pancreatic insufficiency. He had sinus surgery at age 47. Medications include albuterol, ipratropium, fluticasone, dornase alpha, salmeterol, omeprazole, pancreatin and multivitamin. The patient's father had a myocardial infarction at age 37, his mother had coronary artery bypass surgery at 52 years of age, and a brother underwent coronary bypass at age 61. The patient has never smoked and alcohol intake was minimal.

Diagnostic testing demonstrated normal white blood cell, hemoglobin and platelet count. Electrolytes, albumin, protein and glucose were normal with a 5.1% HbA1c. Total cholesterol was 139 mg/dl, LDL 80 mg/dl, HDL 30 mg/dl and triglycerides at 76 mg/dl. Chronic findings with cystic changes were evident on chest radiograph, but with no obvious consolidation. Electrocardiogram revealed sinus rhythm with a left anterior fascicular block and normal ST findings.

Stress thallium testing was subsequently arranged within a week of the follow up visit, with results positive for moderate anteroseptal area of reversible perfusion with a left ventricular ejection fraction of 55%. Subsequent heart catheterization revealed 99% occlusion of the left anterior descending artery with extension into the two diagonal branches, 100% obstruction of the left anterior descending artery at the trifurcation and 70% lesion affecting the first posterior lateral branch of the circumflex artery. The patient was evaluated for possible coronary artery bypass graft; however, because of his current pulmonary status, angioplasty was elected with successful stenting of the left anterior descending artery. On follow up one year out from stent placement, the patient remained asymptomatic with exercise tolerance and pulmonary function returning to baseline.

## Conclusion

This is the first report of a cystic fibrosis patient diagnosed with symptomatic coronary artery disease (CAD) and acknowledges that adult cystic fibrosis patients have indeed survived into the years were coronary artery disease becomes prevalent. Therefore, the likelihood of coronary disease should be included in the diagnostic consideration of persistent dyspnea associated with chest discomfort by applying the same standards used in grading a differential with an anginal presentation in non-CF patients [5].

The patient presented in this case had a Framingham score estimate that predicted a 10-year cardiovascular risk at 4% [6]. However, because of the presence of subtle chest discomfort associated with dyspnea, medical decision making includes an active alternative diagnosis of atypical angina based on two of three positive criteria of exertional symptoms with symptom relief upon resting [5]. Persistent dyspnea may be an occasional finding for a cystic fibrosis patient, and one that typically leads to further work up of a pulmonary etiology; however, cardiovascular disease was additionally considered in this patient.

The typical differential diagnosis of persistent dyspnea in an adult cystic fibrosis patient would include pneumothorax (5% prevalence) [7], atypical mycobacterial pneumonia (15% prevalence) [8], allergic bronchopulmonary aspergillosis (30% prevalence) [9], and cor pulmonale (3% prevalence in association with severe CF pulmonary disease) [10]. Without a history for hemoptysis, atypical mycobacterium and allergic bronchopulmonary aspergillosis would be less likely. Physical exam and chest radiograph did not support either pneumothorax or congestive heart failure. Symptoms could also be explained by this patient's progression of gastroesophageal reflux and/or Barrett's esophagus.

Atypical angina would be the most prevalent differential diagnosis, at 60%, for our patient's clinical presentation of chest pain in the non-CF male patient at this age [5]. The risk of CAD is not typically considered as an active alternative diagnosis for the CF patient; yet with aging, even CF patients will be at risk for CAD. This risk may even be greater given higher prevalence of known risk factors such as diabetes mellitus in the CF versus non-CF patient [11].

Two cases of asymptomatic coronary artery disease have been reported in association with CF with some advanced detail. One case came to diagnosis at autopsy, characterized as generalized atherosclerosis in a 41-year-old female CF patient [homozygous G542X] with diabetes mellitus that died from respiratory failure [12]. Further review of this case revealed a 200 mg/dl averaged cholesterol level, progressive hypertension with biopsy proven nephrosclerosis by 31-years of age, and a diabetic course complicated by gastrointestinal pseudoparesis, retinopathy and neuropathy. In this case, coronary artery disease was an incidental finding on autopsy as she was asymptomatic for myocardial ischemia during her lifetime.

A second case was described in which segmental hypokinesis with grade 2 systolic function was found as an incidental finding on Doppler echocardiography from a prevalence study looking at pulmonary hypertension that included an adult cystic fibrosis patient population [10]. This study identified a 40-year-old diabetic male, diagnosed with asymptomatic coronary artery disease after performing a thallium perfusion scan. Further testing by cardiac catheterization was not reported in this study.

What about other coronary risk factors? Cholesterol and hypertension were not identified risk factors present in our patient. The autopsy case report had several risk factors present, most notably, diabetes and hypertension, but with only a borderline elevated cholesterol level. What is the expected lipid level in a CF patient? The largest lipid study conducted on a CF population reported 134 ± 84 mg/dl triacylglycerol and 138 ± 84 mg/dl total cholesterol values [14]. Only 4% of patients had cholesterol levels >200 mg/dl in this study, with a maximum total cholesterol identified at 240 mg/dl. Serum cholesterol levels can be highly variable in CF patients. Patients with pancreatic insufficiency have low to normal cholesterol levels even with a high fat diet and enzyme supplementation as seen in this case [15]; however, CF patients with pancreatic sufficiency are likely at the same risk as the general population for complications for hyperlipidemia [16]. Another study of aortic atherosclerosis in CF patients found that they have less fatty streaking of the aorta than their weight matched counterparts [17]. Clearly there are other factors that may lead to atherosclerosis than lipid levels; however, applying a clinical practice guide published by the American College of Physicians [5] proved helpful toward diagnosing CAD in this CF patient presenting with dyspnea associated with a chest discomfort.

## Abbreviations

CF – cystic fibrosis; FEV_1 _– forced expiratory volume at 1 second; LDL – low density lipoprotein; HDL – high density lipoprotein; CAD – coronary artery disease;

## Competing interests

The author(s) declare that they have no competing interests.

## Authors' contributions

GMO contributed to the literature review cited in the discussion and editing of the original manuscript. CLF providing medical care for the patient described in this case presentation, and wrote the original manuscript detailing the clinical findings encountered from the clinical assessment. All authors read and approved the final manuscript.

## Pre-publication history

The pre-publication history for this paper can be accessed here:


